# Markers of Aggressiveness in Pituitary Tumors: Update and Perspectives

**DOI:** 10.3390/jcm11216508

**Published:** 2022-11-02

**Authors:** Fabio Bioletto, Alessandro Maria Berton, Nunzia Prencipe, Emanuele Varaldo, Chiara Bona, Silvia Grottoli

**Affiliations:** Division of Endocrinology, Diabetology and Metabolism, Department of Medical Sciences, University of Turin, 10126 Turin, Italy

Pituitary neuroendocrine tumors (PitNETs) are relatively common intracranial neoplasms, potentially originating from various pituitary cell types. Most of these tumors exhibit benign behavior and may be easily cured or controlled via surgery or standard medical treatment. However, in a certain percentage of cases, PitNETs may be characterized by more aggressive local growth, and on rare occasions, by the development of distant metastases. Overall, the exact definition of aggressive PitNET remains debated. According to the 2018 ESE Guidelines [1], this diagnosis should be considered in the case of radiologically invasive tumors with unusually rapid growth rates or in the case of clinically relevant tumor growth despite optimal standard therapies. From a histopathological point of view, in the 2022 WHO classification [2], it is emphasized that the histotype itself may bear a prognostic value; in fact, within each lineage, some PitNET subtypes are associated with a higher risk of aggressive behavior, such as sparsely granulated somatotroph, sparsely granulated corticotroph, silent corticotroph, Crooke cell, male lactotroph, densely granulated lactotroph, and immature PIT1-lineage tumors.

The biological determinants of PitNET aggressive behavior have not yet been fully elucidated. Basic and clinical research in this field, however, is rapidly growing, and this research represents a domain of utmost interest. In fact, a deeper understanding of the pathophysiological mechanisms that underlie PitNET aggressiveness could provide relevant information to inform the clinical management of patients with these tumors; the identification of prognostic markers of aggressiveness could allow for a more accurate prediction of patients’ prognosis and for a better choice of the intensity of follow-up; even more importantly, the identification of reliable markers of response to therapies could be helpful in the optimization and tailoring of treatment choices. Advancements in the radiological, histopathological, genetic, and microenvironment characterization of PitNETs have increased over the last decade, and several markers have been studied and proposed. In this editorial, we summarize some intriguing insights that have been recently published in this complex and heterogeneous research field.

Among histological parameters, tumor proliferation—evaluated by Ki-67, mitotic count, and p53 expression—has historically been considered as a useful index for the identification of PitNETs with potential aggressive behavior, although its exact prognostic value remains controversial. From an anatomical point of view, tumor invasiveness is a predictor of surgical outcome, but also of a patient’s long-term prognosis; indeed, PitNETs with extrasellar extension often invade the cavernous sinus, making their complete resection difficult or impossible; radiologically, PitNETs are generally considered invasive when classified as Knosp grade 3–4 with magnetic resonance imaging (MRI).

In a recent study, Lu et al. [3] retrospectively analyzed data from 903 patients who had undergone transsphenoidal surgery for both functioning and non-functioning PitNETs (NF-PitNETs) and were followed-up after surgery for at least 2 years. Recurrence was defined as the radiological reappearance of a tumor after gross total resection and/or as a re-increase in plasma hormone levels after biochemical remission. Progression was defined as the growth of a residual tumor on MRI and/or as a progressive increase in hormonal values. The authors noted that a Ki-67 expression level ≥3% showed no significant relationship with the degree of invasiveness of the lesion, defined from a histological, surgical, and radiological point of view. Furthermore, although histological invasiveness (i.e., dura mater infiltration) could predict recurrence/progression-free survival (hazard ratio (HR) 1.44, 95% confidence interval (95%CI) 1.14–1.81, *p* = 0.003), its prognostic value was less than that of radiological (HR 5.11, 95%CI 3.98–6.57, *p* < 0.001) or surgical invasiveness (HR 6.40, 95%CI 5.09–8.06, *p* < 0.001).

With respect to NF-PitNETs, other histopathological markers and predictive factors for aggressiveness have been studied. In a recent study, Flores-Martinez et al. [4] systematically evaluated the expression of somatostatin (SST) and dopamine (DA) receptors using both real-time quantitative PCR (RT-qPCR) and immunohistochemistry (IHC) in a large group of patients with NF-PitNETs (*n* = 113); their potential relationship with clinical and molecular aggressiveness features was analyzed, but no relevant association between receptor expression and aggressive features was found. In particular, no significant differences were found between invasion, surgical cure, or tumor regrowth and the expression levels of any SST and DA receptor subtype. Additionally, no significant correlations between SST or DA receptors’ expression and tumor size, age, or sex were observed, neither in the whole cohort, nor in subgroup analyses restricted to gonadotropin-storing and null cell tumors. Finally, no significant correlations between SST or DA receptors’ expression and Ki-67, p53 index level, or E-cadherin IHC score were observed.

Another field of growing interest is represented by the analysis of PitNET microRNA (miRNA) expression profiles. miRNAs are small single-stranded non-coding RNA molecules, which function as post-transcriptional regulators of gene expression through base-pairing with complementary sequences of messenger RNA (mRNA). A dysregulation of the miRNA expression profile has been reported in the pathogenesis of many types of human cancers, with a possible role in tumor initiation and subsequent progression. In recent years, changes in miRNA regulation have also been demonstrated in PitNET cells and correlated with various clinical and biological features.

In a recent study, García-Martínez et al. [5] performed a thorough analysis of the differential expression of miRNAs in silent and functioning corticotroph tumors. The authors collected tissue samples from 47 tumors in the corticotroph line (23 silent and 24 functioning corticotroph tumors). The primary aim of the study was to evaluate the possible role of miRNAs in the silencing mechanisms of these tumors, but secondary analyses were also performed with respect to PitNETs’ size and growth. Interestingly, three of the examined miRNAs (miR-488, miR-200a, and miR-103) were found to be correlated with tumor size, with a significantly higher expression in macroadenomas compared to microadenomas. Possible correlations between miRNAs and invasiveness (defined by Knosp classification) were also assessed; however, no significant differences between invasive and non-invasive tumors could be found. Overall, these data suggest that miRNAs might participate in the pathways of growth of pituitary corticotroph tumors; however, further studies are needed to confirm this result and to more effectively assess their possible future use as prognostic markers or therapeutic targets.

The expression of miRNAs in corticotroph tumors was also examined in another recent study, conducted by Bujko et al. [6]. Twenty-eight patients with functioning and twenty patients with silent corticotroph tumors were included. The primary aim of the study was to evaluate possible differences in miRNA expression according to Ubiquitin Specific Peptidase 8 (USP8) mutational status; this is a subject of significant interest, as USP8 mutations are observed in approximately 30–40% of corticotroph tumors and have been linked to differences in their clinical and biological behavior. In fact, USP8-mutated (USP8mut) corticotroph tumors are more commonly characterized by a smaller size and a higher chance of surgical cure. On the contrary, USP8-wild type (USP8wt) corticotroph tumors are more likely to display aggressive behavior, with a larger tumor size and a lower surgical remission rate. The study by Bujko et al. highlighted some differences between USP8mut and USP8wt tumors in miRNA expression profile; however, these differences were less marked compared to those demonstrated by previous authors in terms of gene and mRNA expression. In fact, only approximately 3.5% of all analyzed miRNAs differed between USP8mut and USP8wt tumors; moreover, less than 10% of the predicted target genes based on miRNA/mRNA correlation analysis showed significantly different expression between these two tumor types. Therefore, based on these data, the differences in miRNA levels, although present, do not appear to play a pivotal role in determining the diversity in gene expression which is observed between USP8mut and USP8wt corticotroph tumors.

In addition to miRNAs, another molecular marker that has been studied in PitNETs in recent years is represented by DNA methylation. An overall decrease in DNA methylation has been observed in many types of human cancers; moreover, it has been proved to correlate with worse patient prognosis and more aggressive cancer growth.

With respect to PitNETs, Rusetska et al. [7] recently performed a study that aimed to determine the role of DNA methylation changes in the biological behavior of nonfunctioning gonadotrophinomas. For this purpose, tumor fragments from 80 patients were collected, and the DNA methylation level of Long Interspersed Nuclear Element-1 (LINE-1) was measured in each. LINE-1 sequences comprise approximately 17% of the human genome, and their methylation status is generally regarded as a surrogate of global DNA methylation. Interestingly, Rusetska et al. found that LINE-1 methylation was correlated with invasive tumor growth; in fact, invasive PitNETs displayed lower LINE-1 methylation levels compared to non-invasive ones. On the other hand, no association was found between LINE-1 methylation and other parameters of aggressiveness, such as the Ki67 index, tumor size, and post-surgical recurrence; however, these latter results may have been hampered by the relatively low sample size.

Finally, increasing attention has been paid to the role of tumor angiogenesis and immune microenvironment, which, in recent years, have been shown to be associated with the aggressiveness of various type of tumors (including melanoma, non-small cell lung cancer, breast cancer, and kidney cancer). These pathways have also been extensively studied as therapeutic targets, with outstanding results in controlling various cancers previously characterized by very poor prognoses. The vascular endothelial growth factor (VEGF)/VEGF receptor (VEGFR) signal is a known potent activator of angiogenesis, related to disease progression and hemorrhage. Moreover, VEGF-A also plays a key role in promoting the development of an immunosuppressive microenvironment, inhibiting dendritic cell maturation and stimulating regulatory T-cell proliferation. However, its exact role in the natural history of PitNETs still needs to be clarified.

In a recent study, Sato et al. [8] evaluated the expressions of VEGF-A/VEGFR and some characteristics of the immune microenvironment on the histopathological tissue of 27 newly diagnosed NF-PitNETs, analyzing their relationship with cavernous sinus invasion as a marker of aggressive behavior. Notably, the expression of VEGF-A and VEGFR1 was higher in NF-PitNETs with cavernous sinus invasion than in those without. Moreover, markers of an immunosuppressive microenvironment, such as the number of tumor-associated macrophages (TAMs) and the expression of programmed death-ligand 1 (PD-L1), which are both induced by VEGF-A, were also associated with the presence of cavernous sinus invasion. No significant differences were observed in mitotic count between the two groups; moreover, the Ki-67 index was <1%, and p53 was immunonegative in all patients.

In conclusion, our understanding of the biological determinants of PitNET aggressiveness has significantly improved in recent years, and several markers have been proposed as possible hallmarks of aggressive behavior. To date, however, most of these results remain investigational, and some difficulties persist in translating them into a routinary clinical setting due to the relative rarity of aggressive forms and to the often-limited availability of the required equipment and assays. Future research will hopefully further improve the characterization and treatment of these tumors. Indeed, aggressive PitNETs are often characterized by a low surgical success rate. Other therapeutic options to avoid further tumor growth are mainly represented by radiotherapy and temozolomide, but sometimes with unsatisfactory results. In this sense, the search for predictors of aggressive behavior can coincide with a deeper characterization of tumors that might be more susceptible to target therapies such as anti-VEGF antibodies, tyrosine kinase inhibitors, and immune checkpoint inhibitors. In the future, larger multicenter studies could facilitate the discovery of novel molecular pathways of tumor growth, which may prompt the identification of new prognostic markers and therapeutic targets and, ultimately, the improvement of patient care.
